# Phosphorescent iridium (III) complex with covalent organic frameworks as scaffolds for highly selective and sensitive detection of homocysteine

**DOI:** 10.3389/fchem.2024.1399519

**Published:** 2024-06-05

**Authors:** Chuti Deng, Juntong Xu, Qi Zhang, Yong Fan

**Affiliations:** ^1^ Department of Chemistry, Fudan University, Shanghai, China; ^2^ Shanghai RNA Cure Biopharma Co., Ltd., Shanghai, China

**Keywords:** Ir(III) complexes, bipyridine derivative, covalent organic framework, emission blue shift, homocysteine sensor

## Abstract

**Introduction:** Developing a convenient and cost-effective platform for detecting homocysteine (Hcy) is of great interest as Hcy has been found to be a biomarker for Alzheimer’s disease, gastric cancer, and other diseases.

**Methods:** In this study, we synthesized five phosphorescent Ir(C^∧^N)_2_(N^∧^N)^+^ compounds (Irn, n = 1–5) with various substituents (-CHO or -CHO/-NH_2_), which were then doped into a covalent organic framework (COF) host via covalent bonding.

**Results and Discussion:** The resulting optimal composites (denoted as Ir4/5@EBCOF) with -CHO/-NH_2_ substituents not only overcame the self-quenching issue of the bare Ir4/5 complexes but also showed rapid, highly selective, and sensitive detection of Hcy, with a limit of detection (LOD) of 0.23 μM and reaction time of 88 s. The sensing mechanism was revealed as the unique cyclization reaction between Ir(III) and Hcy that forms a six-membered ring. During the process, the color changes in the composites can be observed visually. It is expected that these phosphorescent Iridium (III) complexes with COFs will have the potential to serve as promising platforms for detecting thiols.

## 1 Introduction

Thiols, such as cysteine (Cys), homocysteine (Hcy), and glutathione (GSH), play important roles in biological and physiological activities, serving as protective agents in the form of antioxidants and free radical scavengers (Yin et al., 2017). Maintaining sufficient thiol levels in the human body is thus crucial for good health. Extant studies have established solid links between mental diseases and abnormal levels of thiols within the human body (Seshadri et al., 2002; Behera et al., 2017; Hasan et al., 2019). For example, patients with Alzheimer’s disease, cardiovascular issues, or osteoporosis often have increased Hcy levels in their serum. High Hcy levels can result in elevated risks for gastric cancer and other health problems (Behera et al., 2017; Hasan et al., 2019). There are several traditional analytical methods for Hcy quantification, such as radioenzyme analysis (RIA), high-performance liquid chromatography (HPLC), electrochemistry, capillary electrophoresis (CE), and colorimetry (Pasas et al., 2002; Sawula et al., 2008; Leesutthiphonchai et al., 2011; Baron and Sochor, 2013; Alam et al., 2019). However, the use of radioactive adenosine leads to environmental pollution and requires expensive antibodies. Instrumental analyses also have certain inherent limitations, including tedious sample preparation, complicated chemical modifications, and lengthy analysis times. Therefore, it is of great interest to develop a convenient, simple, and cost-effective method for detecting Hcy content in practical scenarios.

Optical sensing has been recommended as a potential method for detecting Hcy owing to its advantages of easy-to-perform operation, low requirement of equipment, and instant results compared to the approaches (Niu et al., 2015). Researchers have explored various optical sensing probes and platforms that have specific reactions in the presence of Hcy, such as nucleophilic substitution, Michael addition, cyclization with aldehydes, and cleavage reactions (Lee et al., 2012; Liu et al., 2015; Niu et al., 2015; Nehra et al., 2020). These reactions on probes cause changes in the optical properties, such as the emission lifetime (τ), emission intensity, and emission quantum yield (ϕ). Among these, there is an interesting cyclization reaction between Hcy and the aldehyde (-CHO) group in the probe that forms a six-membered ring accompanied by emission intensity variations and red-/blue-shifted wavelengths. For instance, xanthene derivatives containing a -CHO group have been reported by Strongin and coworkers to have the ability to optically recognize Cys and Hcy (Rusin et al., 2004). Wong and coworkers have reported Hcy probes based on aldehyde-modified triphenylamine and carbazole with two-photon absorption behaviors (Yang et al., 2012). A series of Ir(III) compounds containing aldehydes have been reported for Hcy detection and compared carefully (Gao et al., 2017; Wang et al., 2018); their findings suggest that Ir(C^∧^N)_2_(NN)^+^ compounds (C^∧^N is a cyclometalating ligand and N^N is 2,2′-bipyridine) with aldehyde-containing ligands can be developed as Hcy probes since these compounds show significant changes in their emission wavelengths and ϕ after cyclization reactions with Hcy, which can be modulated as optical sensing signals (Ma et al., 2011).

Although Ir(C^∧^N)_2_(NN)^+^ compounds have shown attractive Hcy sensing potential, there is one issue with the self-quenching effects of these probes containing large fused polynuclear rings that must be addressed when dispersed in aqueous media, which limits their sensitivities (Li et al., 2015; Zhu et al., 2019). Owing to the strong interactions between the long-lived excited states, their energies are easily quenched and exhausted by the surrounding molecules, resulting in reduced ϕ values or emission intensities. Research on such self-quenching behaviors has been reported in the incorporation of Ir(III)-based materials into organic light-emitting devices (Kawamura et al., 2006; Song et al., 2023; Temram et al., 2023). One universal solution to the abovementioned problems to date is to disperse Ir(III) compounds in an appropriate host material. There have been many efforts focusing on the development of porous materials as promising supporting matrices for metal complexes to prolong the detection lifetimes of catalytic systems (You et al., 2020; Li et al., 2022; Tselekidou et al., 2023; Wang et al., 2023).

**SCHEME 1:**
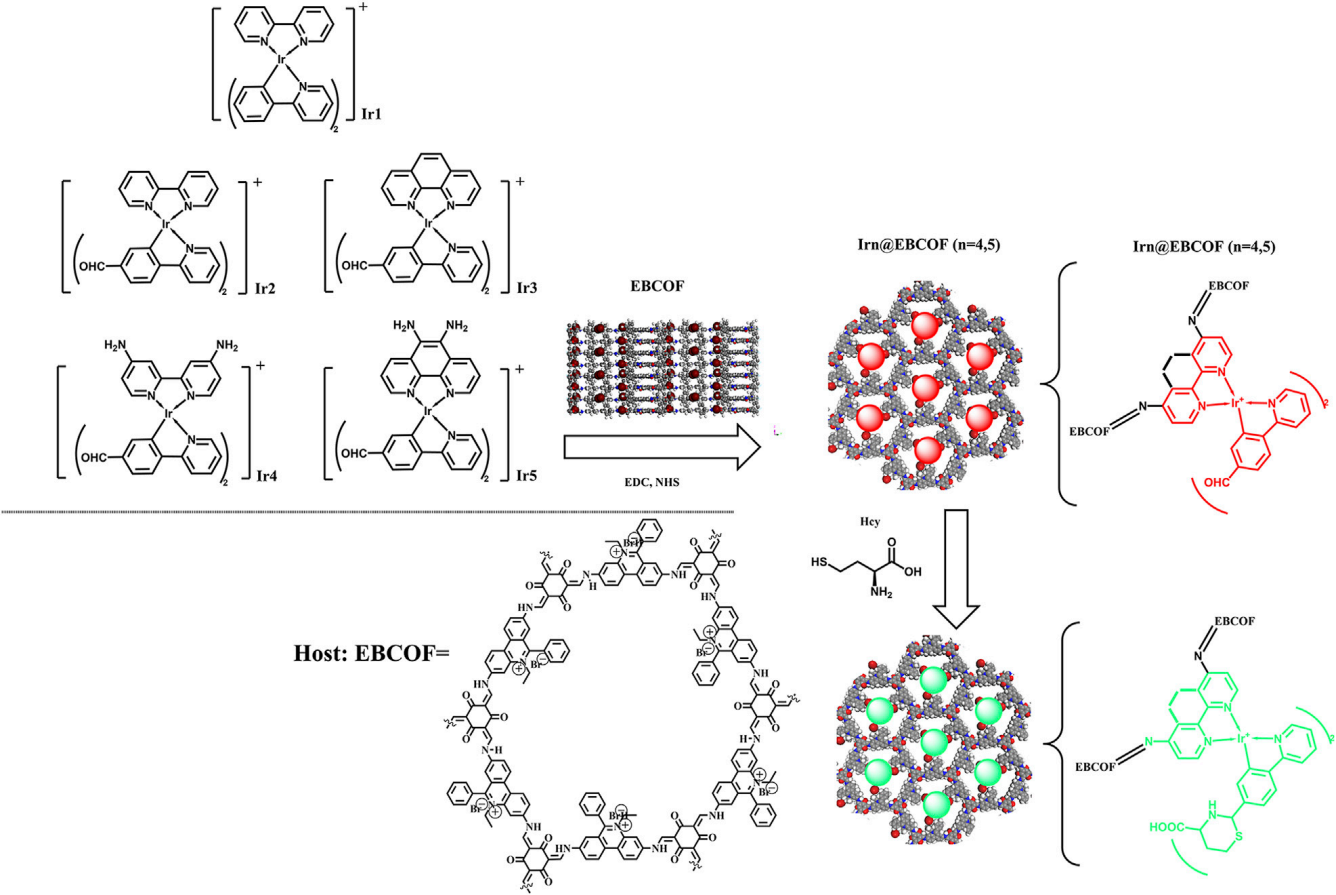
Synthesis route and working mechanism of Irn (n = 1–5) probes and Irn@EBCOF (n = 4, 5) composite samples.

In the present work, we synthesized a covalent organic framework (COF)-based host (denoted as EBCOF) that had a consistent, adjustable, and reliable pore structure. A series of Ir(C^∧^N)_2_(N^∧^N)^+^ compounds (Ir1, Ir2, Ir3, Ir4, and Ir5) with various substituents, including -CHO group and electron-donating -NH_2_ groups, were synthesized and tested as Hcy probes, as shown in Scheme 1. These Ir(III) compounds were systematically analyzed and compared. The optimal probes with sensitive changes in their emission intensity and wavelength for Hcy were doped into the EBCOF host via covalent bonding. The resulting composites (denoted as Irn@EBCOF, n = 4, 5) were tested for Hcy sensing, and their sensing performances were analyzed in detail. During the detecting process for Hcy, the Ir5@EBCOF probe achieved a low limit of detection (LOD) of 0.23 μM, high sensitivity of 1.0106 μM^-1^, quick response of ∼88 s, and a trend for visible color change from red to green to naked eyes under ultraviolet light.

## 2 Materials and methods

### 2.1 Reagents and equipment

The molecular structures of Ir1, Ir2, Ir3, Ir4, and Ir5, along with the synthesis strategy and working mechanism of Irn@EBCOF (n = 4, 5), are shown in Scheme 1. The chemicals and reagents used in this work were obtained commercially: 2.2′-bipyridine; [2,2'-bipyridine]-4,4'-diamine; 1,10-phenanthroline; 1,10-phenanthroline-5,6-diamine; 2-phenylpyridine; 4-(pyridin-2-yl)benzaldehyde; IrCl_3_•3H_2_O; phosphate-buffered saline (PBS); 2,4,6-trihydroxybenzene-1,3,5-tricarbaldehyde; dioxane; mesitylene; aqueous acetic acid; 3,8-diamino-5-ethyl-6-phenylphenanthridin-5-ium bromide. The emission ϕ values of Irn (n = 1–5) were determined following a reported method with a standard reference of quinine sulfate (in 1.0 M sulfuric acid, emission quantum yield (ϕ) = 0.546) (Zhang and Li, 2009). The initial geometry was obtained from an Irn single crystal and then optimized using MOPAC (version 22.0.6) with the PM6 method.

### 2.2 Synthesis of Irn compounds (n = 1–5)

The five Ir(C^∧^N)_2_(N^∧^N)^+^ compounds (Ir1, Ir2, Ir3, Ir4, and Ir5) were synthesized by a classic method (Liu et al., 2017). First, the Ir dimer was synthesized as below. A mixture containing IrCl_3_•3H_2_O (0.68 mmol), C^∧^N ligands (1.36 mmol, 2-phenylpyridine/4-(pyridin-2-yl)benzaldehyde), 2-ethoxyethanol (20 mL), and H_2_O (5 mL) was stirred and heated at 105°C for 48 h in an N_2_ atmosphere. After natural cooling, cold water (5 mL) was added to obtain the solid product. The crude product was flushed with ethanol and hexane, dried as an Ir dimer, and used in the next step. A mixture containing the Ir dimer (0.3 mmol), N^∧^N ligands (0.75 mmol, 2,2′-bipyridine/2,2′-bipyridine/1,10-phenanthroline/1,10- phenanthroline-5,6-diamine), dichloromethane (15 mL), and methanol (15 mL) was stirred at 45°C for 12 h in an N_2_ atmosphere. The residual solvent was removed by evaporation under reduced pressure, and the solid product was dispersed in diethyl ether (5 mL) and stirred for 30 min. The crude product was purified on an Al_2_O_3_ column (FCP 100-200) with petroleum ether and acetic ether (v:v = 30:1) as the eluent. The synthesis details and characterizations can be found in the Supplementary Material.

### 2.3 Synthesis of EBCOF and Ir4/5@EBCOF

The porous EBCOF host was synthesized according to a reported procedure (Chen et al., 2020). A mixture of 2,4,6-trihydroxybenzene-1,3,5-tricarbaldehyde (1.5 mmol), dioxane (5 mL), mesitylene (5 mL), aqueous acetic acid (1 mL, 6 M), and 3,8-diamino-5-ethyl-6-phenylphenanthridin-5-ium bromide (1.5 mmol) was stirred at ambient conditions for 15 min. Then, this mixture was degassed and transferred into a Pyrex tube. After heating at 120°C for 3 days, the solid product was collected, washed using tetrahydrofuran/ethanol (v:v = 1:1), and dried in vacuum overnight (yield: 52%).

Ir4/5@EBCOF was synthesized by doping Ir4 and Ir5 into the EBCOF synthesized above via covalent grafting (Ma et al., 2016; Zhao et al., 2021). EBCOF (3 mmol) was dispersed in ethanol (50 mL) and stirred for 30 min; Ir4 or Ir5 (3 mmol, excess amount vs EBCOF) was dissolved in dehydrated dimethylformamide (DMF, 10 mmol) and added dropwise into the above EBCOF suspension. The resulting mixture was stirred at ambient conditions for 18 h. The resulting solid sample was centrifuged, washed with ethanol, and dried in vacuum overnight.

### 2.4 Sensing performance measurements

The Hcy sensing performance of Irn@EBCOF (n = 4, 5) was evaluated as follows. First, the Irn@EBCOF stock solution (pH = 7.0, 2.5 mg/mL) in PBS (100 mL, pH = 7.5, 0.01 M) was prepared. To each portion of the Irn@EBCOF stock solution, a controlled amount of the Hcy standard solution or human serum was added, and diluted with PBS until the Irn@EBCOF concentration decreased to 1 mg/mL. The sample was then treated in an ultrasonic bath for 10 min before obtaining the emission spectrum under an excitation wavelength of 605 nm (5 nm × 5 nm) at a temperature of 25°C. Each spectrum was repeated for thrice so that a mean value could be calculated.

## 3 Results and discussion

### 3.1 Irn (n = 1–5) compounds: performance comparisons and probe selection

#### 3.1.1 Molecular design and geometric structures

The target molecular structures of Irn (n = 1–5) are shown in Scheme 1. Despite having a basic molecular formula of [(C^∧^N)_2_Ir(N^∧^N)]Cl, these structures were divided into three groups according to their ligands. The first group (Ir1) was introduced as the reference group without any -CHO group. Ir2 and Ir3 with a -CHO group in their C^∧^N ligands comprised the second group. The third group consisted of Ir4 and Ir5, with one -CHO group each in their C^∧^N ligands and two -NH_2_ groups each in their N^∧^N ligands.

**FIGURE 1:**
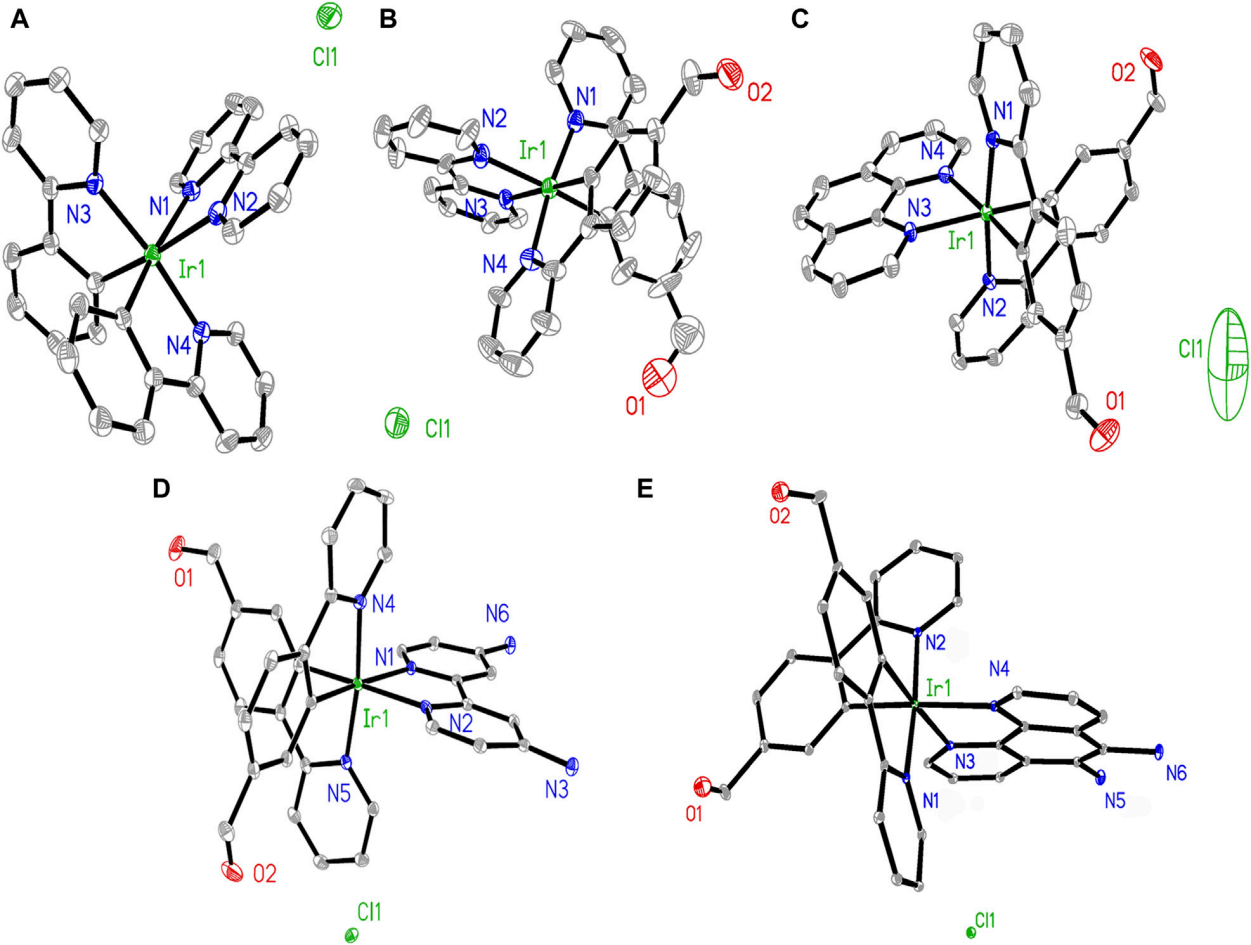
Geometric structures of **(A)** Ir1, **(B)** Ir2, **(C)** Ir3, **(D)** Ir4, and **(E)** Ir5.

Comparisons were performed between these three groups to determine clues for evaluating their Hcy sensing performances. The -CHO group has been reported to be sensitive and is known to form a six-membered ring with Hcy; the -NH_2_ group is able to facilitate this cyclization reaction (Rusin et al., 2004). The single-crystal structures of Ir1, Ir2, Ir3, Ir4, and Ir5 are shown in Figure 1. Two C^∧^N and one N^∧^N ligands form a typical octahedral coordination field for the central Ir(III) ion, which is consistent with literature (Zhang et al., 2009). There are no observed signs of coordination between the Ir(III) and Cl^−^ ions, indicating that the Cl^−^ ions act only as counterions.

#### 3.1.2 Photophysical properties

**FIGURE 2:**
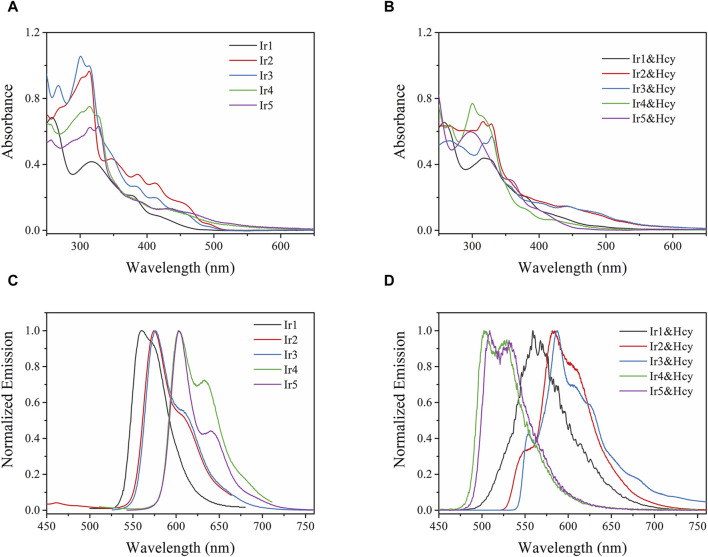
**(A,B)** Absorption and **(C,D)** emission spectra of Irn in DMF (1 μM) without and with homocysteine (Hcy) addition (5 equiv.).

We first compared the absorption and emission spectra of the Irn compounds (1 μM) in DMF (Figure 2). Their key spectroscopic parameters are listed in Table 1. Owing to their similar molecular structures, the Irn compounds show similar absorption bands, and the absorption spectra are composed of intense absorption bands ranging from 250 nm to 335 nm and moderate ones ranging from 335 nm to 515 nm (Figure 2A). The intense absorption bands have been reported as ligand π→π* transitions, while the moderate absorption bands belong to metal-to-ligand charge transfer (MLCT) transitions (Liu et al., 2017). The introduction of the electron-withdrawing group -CHO tends to move the absorption edge (λ_edg_) of Ir2/Ir3 (515 nm) toward longer wavelengths (red shift) than that of Ir1 (490 nm). Similarly, a larger wavelength redshift of 90 nm was observed for Ir4/Ir5 (592 nm) by the introduction of both -CHO and electron-donating -NH_2_ groups.

**TABLE 1:** Spectroscopic parameters of Irn (n = 1–5) in DMF (1 μM) with and without Hcy.

Irn	λ_edg_ ^a^ (nm)	λ_em_ ^b^ (nm)	FWHM (nm)^c^	ϕ (%)^b^	τ (μs)^c^	k_r_ (s^-1^)×10^4^	k_nr_ (s^-1^)×10^4^
Ir1	490/491	588/559/560	44	0.03/11.5/6.2	3.39	3.39	26.11
Ir2	515/592	611/574/584	50	0.02/7.5/6.4	2.73	2.74	33.78
Ir3	515/589	614/576/588	53	0.04/7.4/6.1	3.06	2.41	30.23
Ir4	592/498	619/602/503	57	0.02/11.7/13.3	3.38	3.46	26.15
Ir5	592/494	620/603/509	30	0.03/11.2/13.1	3.41	3.28	26.02

^a^ For data in the format of “AAA/BBB”, AAA: without Hcy, BBB: Hcy (5 equiv.).

^b^ For data in the format of “CCC/DDD/EEE”, CCC: in solid, DDD: in solution without Hcy, EEE: in solution with Hcy (5 equiv.).

^c^ In solution without Hcy, the weighted average lifetime of biexponential decay (τ)=(A1τ_1_^2^ + A2τ_2_^2^)/(A1τ_1_+A2τ_2_).

Owing to the aggregation-induced quenching effect (*ϕ* values <0.04%) in the solid state (Zhang et al., 2009), all Irn compounds show little or no emissions (Table 1). However, after being dispersed in solution, their quantum yields (>7%) are enhanced by two orders of magnitude. Each emission spectrum is also composed of a major band and a shoulder band, which is a characteristic of MLCT-based Ir(III) emission (Liu et al., 2017). With the introduction of -CHO or -NH_2_ and -CHO groups, the Irn (n = 2/3/4/5) emissions show redshifts compared to that of Ir1 (559 nm) (Table 1). It should be noted that the full-width at half maximum (FWHM) value of Ir5 (30 nm) is less than those of the other Irn compounds (ranging from 44 to 57 nm), indicating higher color purity (Fan et al., 2023). We next measured the decay lifetimes of the Irn (*n* = 1–5) compounds (Table 1), all of whom presented biexponential decay modes. The long-lived component of the decay lifetime is attributed to the decay process of the MLCT excited state, and the short-lived component is attributed to that of the ligand π→π* excited state, consistent with the findings in literature (Zhang et al., 2009). The radiative transition rate constant of the excited state (k_r_) and non-radiative transition rate constant of the excited state (k_nr_) of Irn were calculated using Eqs. (1, 2) and listed in Table 1. Compared with Ir1, the decrease in the electron density of Ir2/3 caused by introduction of the electron-withdrawing group -CHO significantly improves the value of k_nr_ and reduces the value of k_r_, consistent with the short lifetime (2.73–3.06 s) and low ϕ (7.4%–7.5%) (Xia et al., 2023). Similarly, the electron-donating -NH_2_ group further improves the emissive probability since it increases the electron density of the excited state.

$$\phi =\frac{k_{r}}{k_{r}+k_{nr}}$$ (1)

$$\tau =\frac{1}{k_{r}+k_{nr}}$$ (2)

The absorption and emission spectra of Irn were recorded in the presence of Hcy (5 equiv.) (Figures 2B, D). The absorption spectrum of Ir1 remains unchanged, with no detectable spectral shift or new bands after adding Hcy, indicating that the molecular structure of Ir1 is preserved. As for Ir2 and Ir3 with the -CHO group in their ligands, slight emission redshifts (∼10 nm) accompanied by emission quenching (from 7.5%–6.4% and 7.4%–6.1%, respectively) are observed after adding Hcy. In addition, an additional shoulder band peaking at 554 nm was observed. For Ir4 and Ir5, the absorption spectra blue-shifted from ∼592 nm to ∼498 nm in the presence of Hcy. Correspondingly, their emission peaks also blueshifted from 602 to 503 nm and 603 to 509 nm, with the corresponding emission yields increasing slightly from 11.7%–13.3% and 11.2%–13.1%, respectively. The spectral changes of Ir2/Ir3/Ir4/Ir5 suggest interactions between their -CHO groups and Hcy, which make them effective sensing probes for Hcy.

#### 3.1.3 Sensing mechanism

Among the Irn compounds, Ir4 and Ir5 show the most sensitive changes in emission intensities and wavelength shifts upon Hcy addition (∼100 nm) (Table 1). As mentioned above, the absorption spectrum (Figure 2A), emission spectrum (Figure 2B), and decay process of Ir4/5 compounds are consistent with the MLCT mechanism. To better understand the photophysical properties, the frontier molecular orbitals (FMOs) of the Ir4/5 complexes were calculated using time-dependent density functional theory (TD-DFT) and listed in Table 2. Orbital analyses revealed that for the Ir4/5 complexes, the highest occupied molecular orbitals (HOMOs) are composed of the iridium center and phenyl parts of the cyclometalated ligands, whereas the lowest unoccupied molecular orbitals (LUMOs) are located mainly on the bipyridine derivative ligands. The HOMO→LUMO transitions could contribute to the [dπ(Ir) → π_N^∧^N_^*^]^3^MLCT, with some mixing of the ^3^[π_C^∧^N_ → π_N^∧^N_^*^] ligand-to-ligand charge transfer (^3^LLCT). For Ir4/5-Hcy, the HOMOs are located on the iridium center and one newly generated six-membered thiazolidine ring, while the LUMOs reside on the bipyridine derivative ligands, indicating that their emissions are derived mainly from [π_C^∧^N_ → π_N^∧^N_^*^]^3^LLCT and [dπ(Ir) → π_N^∧^N_^*^]^3^MLCT. Therefore, the electronic transitions of these Ir(III) complexes have an MLCT character, and the formed thiazolidine ring has better electron-donating ability than the aldehyde group, which could increase the MLCT transition energy, leading to a blue shift.

**TABLE 2:** HOMO and LUMO distributions of Ir4, Ir4-Hcy, Ir5, and Ir5-Hcy.

Complex	HOMO	LUMO
Ir4	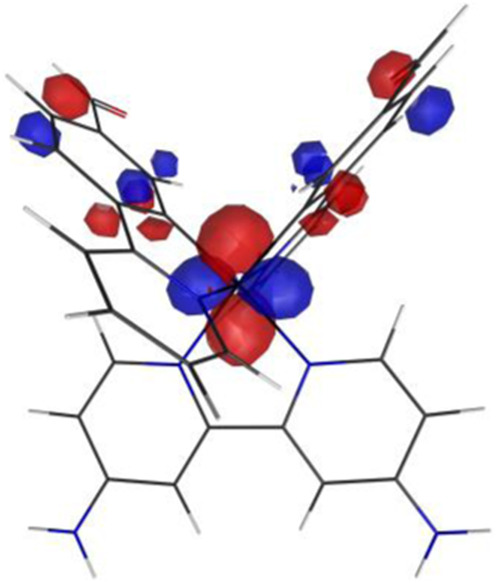	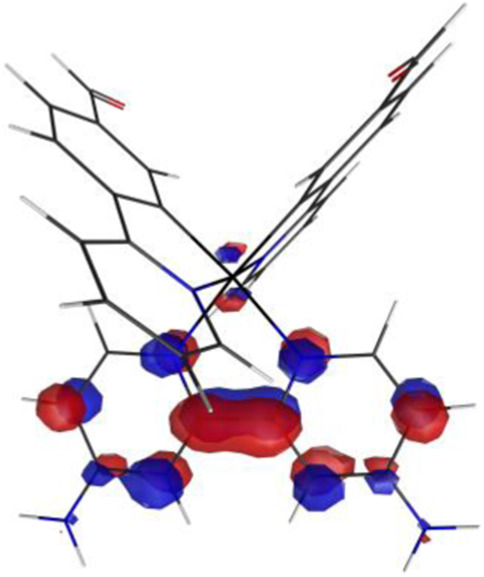
Ir4-Hcy	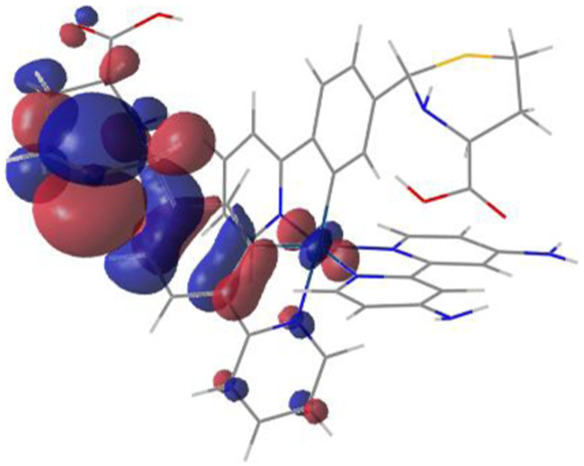	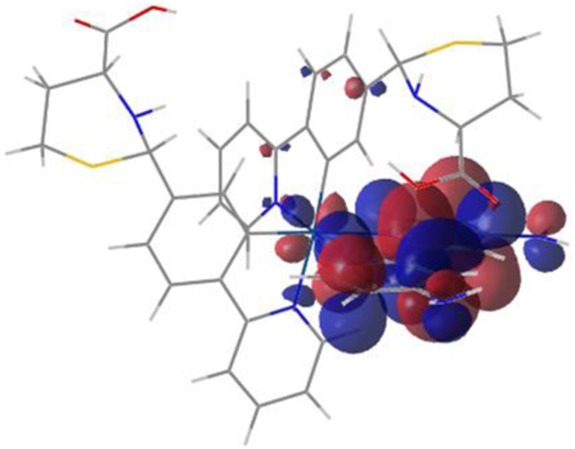
Ir5	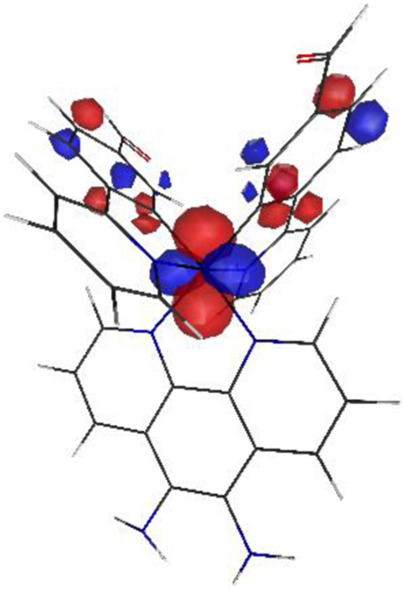	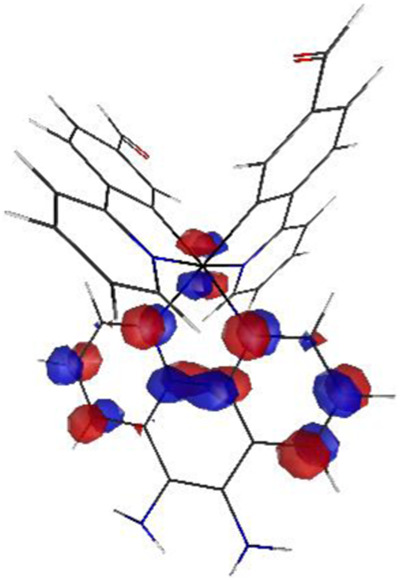
Ir5-Hcy	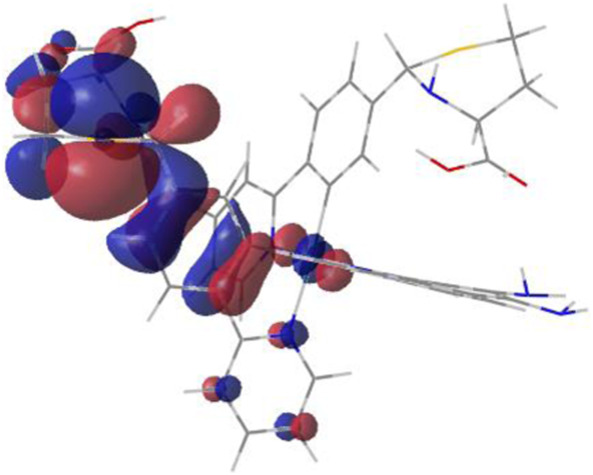	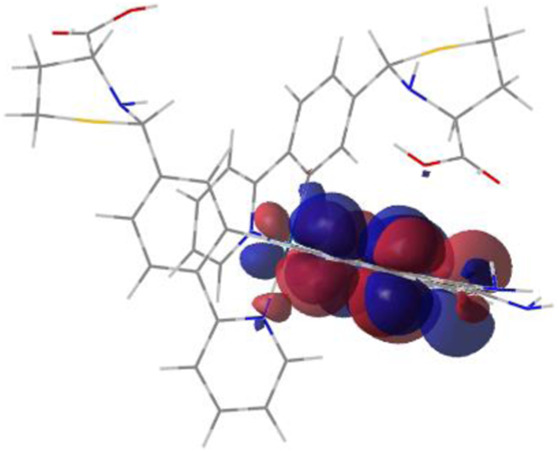

To investigate the sensing mechanism, a nuclear magnetic resonance (NMR) titration was carried out on Ir4 (the results of Ir5 are similar to those of Ir4). As the Hcy concentration increases from 0 to 2 equiv., the chemical shift signal from the H of the -CHO group (9.94 ppm) decreases significantly (Supplementary Figure S1). Meanwhile, chemical shifts are seen for the -NH- (5.24 ppm) and -N-CH-S- (5.11 ppm) groups that become stronger (Supplementary Figure S1). These results indicate that the cyclization between the -CHO group and Hcy is consistent with previous reports (Gao et al., 2017; Wang et al., 2018). The reactions of Ir4/5 on Hcy were further supported by positive-ion electrospray ionization high-resolution mass spectra (ESI-MS) (Zhang et al., 2019) (Supplementary Figure S2). There are strong signals of 978.2 and 1001.2 for the complex [Ir4+2Hcy-PF_6_]^+^ and complex [Ir5+2Hcy-PF_6_]^+^, respectively. Relatively weak signals corresponding to [Ir4+Hcy-PF_6_]^+^ and [Ir5+Hcy-PF_6_]^+^ are also observed at 861.2 and 884.2, respectively. The biform and monoform adducts observed in the ESI-MS indicate that the reactions are stepwise processes.

### 3.2 Characterization on Irn@EBCOF (n = 4, 5)

**FIGURE 3:**
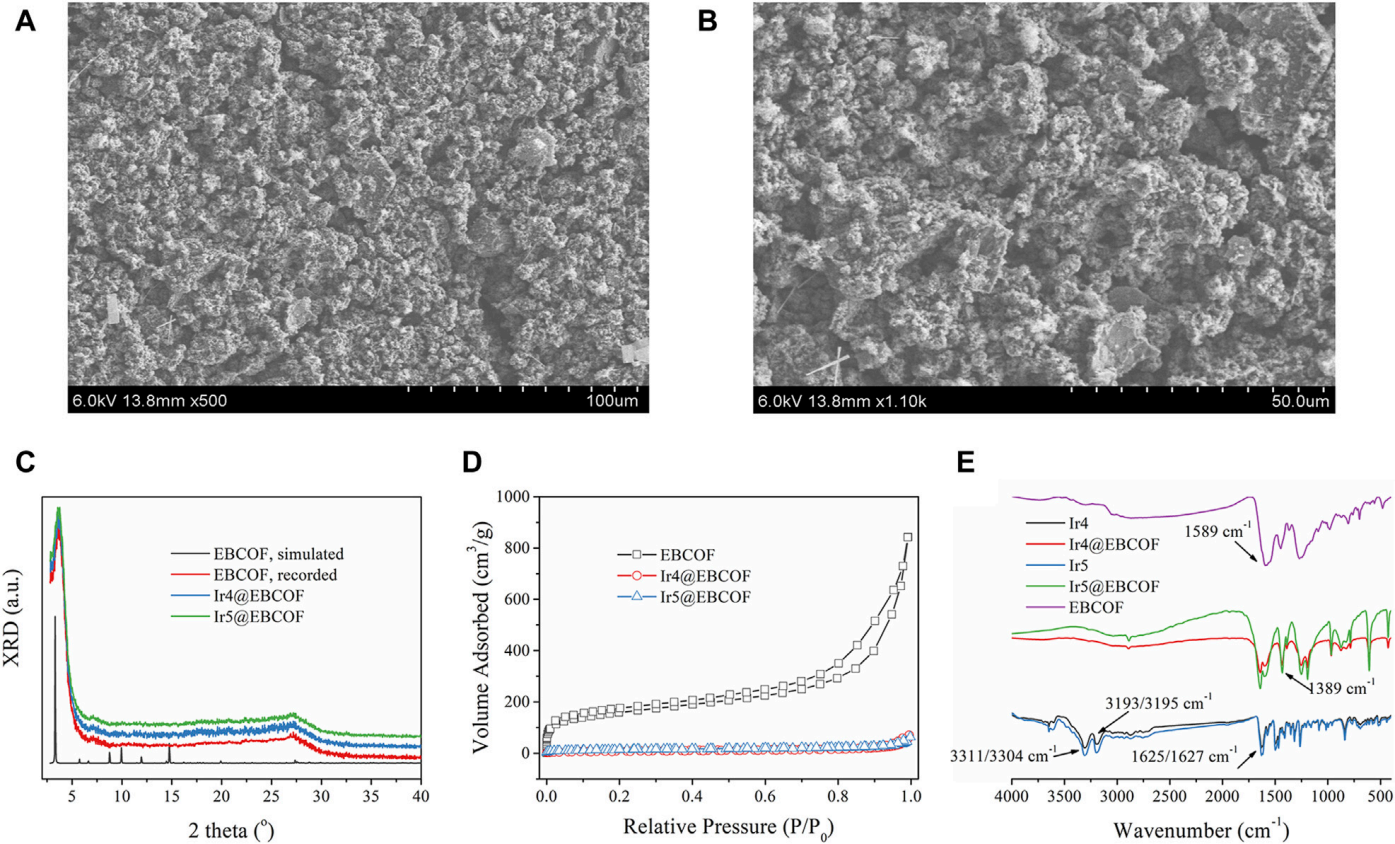
Scanning electron microscopy (SEM) images of **(A)** Ir4@EBCOF and **(B)** Ir5@EBCOF. **(C)** X-ray diffraction curves of EBCOF (simulated and recorded), Ir4@EBCOF, and Ir5@EBCOF. **(D)** N_2_ adsorption/desorption isotherms of EBCOF, Ir4@EBCOF, and Ir5@EBCOF. **(E)** IR spectra of EBCOF, Ir4, Ir5, Ir4@EBCOF, and Ir5@EBCOF.

To further overcome aggregation-induced self-quenching and attenuate the background noise, the optimal Ir4/Ir5 molecules were dispersed in the EBCOF host owing to its suitable pore size (∼2 nm) (Supplementary Figure S3), along with the active group (C=O) to be bonded with Ir4 and Ir5 (Chen et al., 2020). The resulting composites Ir4/5@EBCOF have rough and fluffy surfaces without anisotropy (Figures 3A, B). Their X-ray diffraction (XRD) curves show no new peaks or spectral shifts over those of the as-synthesized EBCOF (Figure 3C), indicating that EBCOF was preserved well after loading with Ir4 or Ir5. We next performed N_2_ adsorption/desorption isotherm measurements (Figure 3D). A type-I isotherm is observed for the EBCOF with a maximum N_2_ uptake value of 840 cm^3^/g (Chen et al., 2020). As for the Ir4/5@EBCOF samples, the maximum N_2_ uptake values decreased significantly to ∼70 cm^3^/g. This result suggests that the pores in the EBCOF are mostly occupied by the probe Ir4 or Ir5 compounds.

To further demonstrate the reliable bonding that enables heavy molecular loading, we compared the IR spectra of EBCOF, Ir4/Ir5, and Ir4/5@EBCOF. As shown in Figure 3E, EBCOF has a simple infrared (IR) spectrum with a C=O vibration peak at 1589 cm^-1^. Ir4 shows characteristic IR bands from the -NH_2_ group, peaking at 3311 cm^-1^ and 3193 cm^-1^. The sharp IR peak at 1625 cm^-1^ is attributed to the vibration from the -CHO group. Similar IR bands are also observed for Ir5, peaking at 3304 cm^-1^, 3195 cm^-1^, and 1627 cm^-1^. The IR peak for Ir4/5@EBCOF from the C=O vibration at 1589 cm^-1^ is preserved but weakened, and no IR peaks are observed from -NH_2_. However, a new IR peak is formed at 1389 cm^-1^, which is assigned to the C=N vibration. These results confirm the covalent bonding between the probe (-NH_2_) and EBCOF (C=O).

### 3.3 Hcy sensing performance of Ir4/5@EBCOF

#### 3.3.1 Emission spectra and working calibration equations on Hcy

**FIGURE 4:**
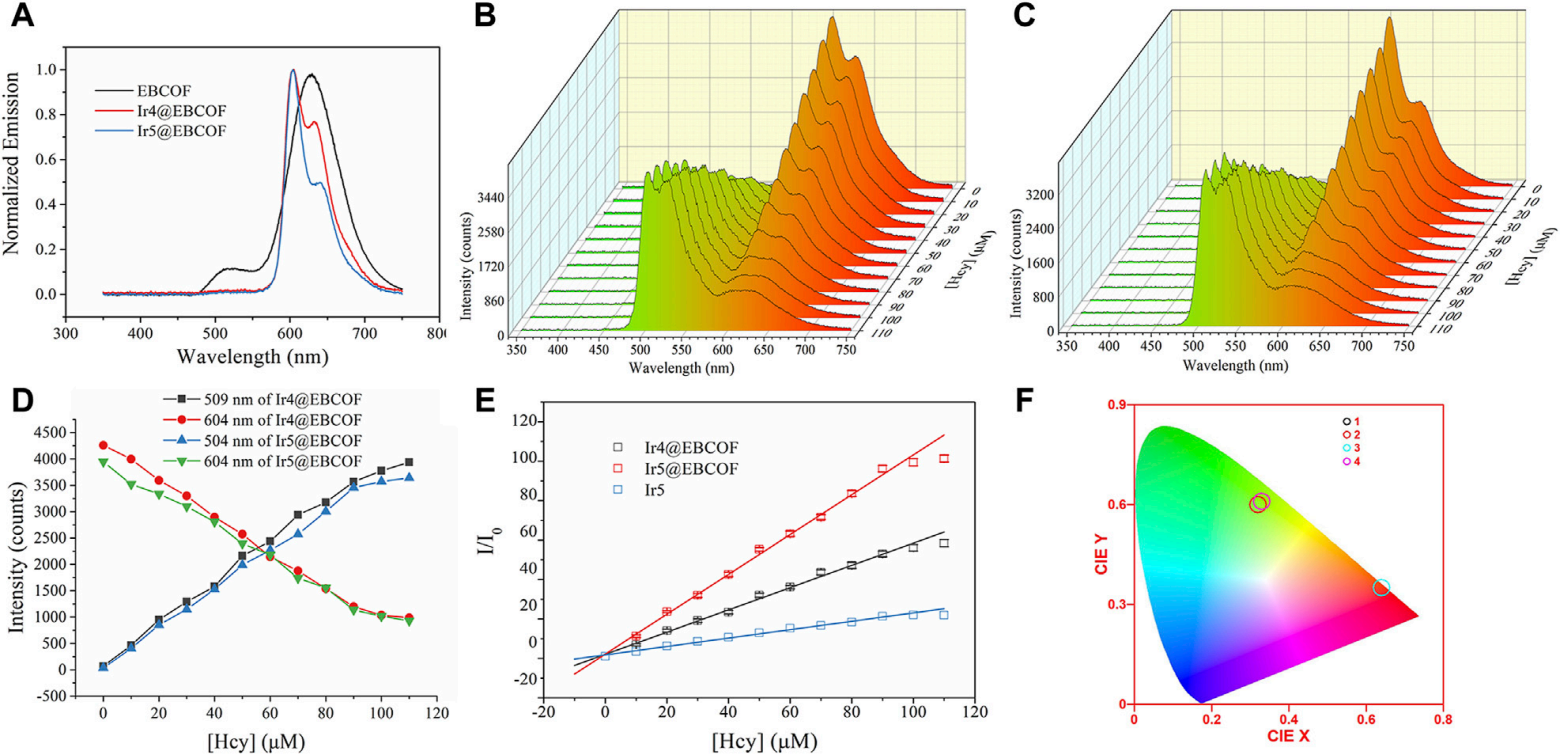
**(A)** Emission spectra of EBCOF, Ir4@EBCOF, and Ir5@EBCOF. Emission spectra of **(B)** Ir4@EBCOF and **(C)** Ir5@EBCOF in PBS (2.5 mg/mL) for increasing Hcy concentrations. **(D)** Corresponding emission intensity variations of Ir4@EBCOF and Ir5@EBCOF. **(E)** Stern–Volmer plots of Ir4@EBCOF, Ir5@EBCOF, and Ir5 for increasing Hcy concentrations, where the lines are the fitting lines. **(F)** CIE coordinates of Ir4@EBCOF (1: without Hcy; 2: [Hcy] = 110 μM) and Ir5@EBCOF (3: without Hcy; 4: [Hcy] = 110 μM).

A spectroscopic analysis of Ir4/5@EBCOF with increasing Hcy concentration was performed to evaluate the Hcy sensing performance. As shown in Figure 4A, in the absence of Hcy, characteristic Ir(III) emissions are observed for Ir4/5@EBCOF: 604 nm (major peak) and 632 nm (shoulder peak) for Ir4@EBCOF; 604 nm (major peak) and 640 nm (shoulder peak) for Ir5@EBCOF; these are accompanied by ∼2 nm wavelength redshifts compared to the bare Ir4/5 molecules (Figure 2C, peaking at 602 nm for Ir4 and 603 nm for Ir5). The reasons for this may be explained by the stabilization or solvation effects (Yang et al., 2011).

As the Hcy concentration increases from 0 to 110 μM, the emission intensities of Ir4@EBCOF at 504 nm and Ir5@EBCOF at 509 nm continue to increase until saturation. Meanwhile, the emission intensity of Ir4/5@EBCOF at 604 nm decreases correspondingly, with the residual emission still having an intensity as high as 1000 counts (Figures 4B–D). This may be attributed to the contribution from the bare EBCOF (627 nm, Figure 4A). To avoid this emission interference, green emission (504 nm for Ir4@EBCOF and 509 nm for Ir5@EBCOF) was selected for evaluating the sensing sensitivity based on Stern–Volmer analysis (Lei et al., 2006). The emission of Ir5 (509 nm) was also analyzed to explore the effect of the EBCOF on Hcy sensing performance, as given by Eq. (3):

$$\frac{I}{I_{0}}=C+K_{sv}\left[Hcy\right]$$ (3)

where I and I_0_ indicate steady emission intensities at 504 nm or 509 nm with and without the addition of Hcy, respectively; C is a constant, K_sv_ is the Stern–Volmer coefficient also known as the sensitivity, and [Hcy] is the concentration of Hcy. As shown in the fitting plots of Figure 4E, linear calibration curves are observed for both Ir4/5@EBCOF and Ir5 samples, with their working range, fitting parameter, LOD (3σ/N), and limit of quantification (LOQ, 10σ/N) (Magnusson and Örnemark, 2014) values summarized in Table 3. The LOD and sensitivity values are determined as 0.44 μM and 0.5641 μM^-1^ for Ir4@EBCOF, and 1.18 μM and 0.2127 μM^-1^ for Ir5, respectively. In the comparison, Ir5@EBCOF shows the best sensing performance, with LOD and sensitivity values of 0.23 μM and 1.0106 μM^-1^, respectively. The much better detection performance of Ir4/5@EBCOF compared to that of Ir5 implies good dispersion of EBCOF.

**TABLE 3:** Detailed sensing parameters of Ir4@EBCOF, Ir5@EBCOF, and Ir5.

Parameters	Ir4@EBCOF	Ir5@EBCOF	Ir5
I_calibration_	2.037 + 0.5641 [Hcy]	2.186 + 1.0106 [Hcy]	1.785 + 0.2127 [Hcy]
*R* ^2^	0.997	0.998	0.992
LOD (μM)	0.44	0.23	1.18
LOQ (μM)	1.47	0.77	3.93
Working range (μM)	1.47–100	0.77–100	3.93–100
Accuracy (serum)^a^	11.9/12.5 (105.04%)	12.0/12.7 (105.83%)	/
Accuracy (serum+30 μM)^a^	41.8/42.2 (100.96%)	42.1/42.4 (100.71%)	/
Accuracy (serum+60 μM)^a^	71.6/70.7 (98.74%)	71.9/70.9 (98.61%)	/
Accuracy (serum+90 μM)^a^	101.5/97.5 (96.06%)	101.5/97.6 (96.16%)	/

^a^ Serum sample or extra Hcy addition; data before “/“: determined by HPLC method and used as reference; data after “/“: determined by Ir4/5@EBCOF; data in “()”: recovery%.

It should be noted that the opposing changes between the green and red emissions of Ir4/5@EBCOF can be viewed based on color. In the absence of Hcy, the CIE color coordinates of Ir4@EBCOF (0.64, 0.35) and Ir5@EBCOF (0.64, 0.35) fall within the red region. For a Hcy concentration of 110 μM, the CIE color coordinates of Ir4@EBCOF (0.32, 0.60) and Ir5@EBCOF (0.33, 0.61) move toward the green region (Figure 4F). Based on these characteristics, Ir4/5@EBCOF are also expected to be useful as visual sensors for Hcy.

#### 3.3.2 Sensing selectivity of Ir4/5@EBCOF and sensing performance in serum

**FIGURE 5:**
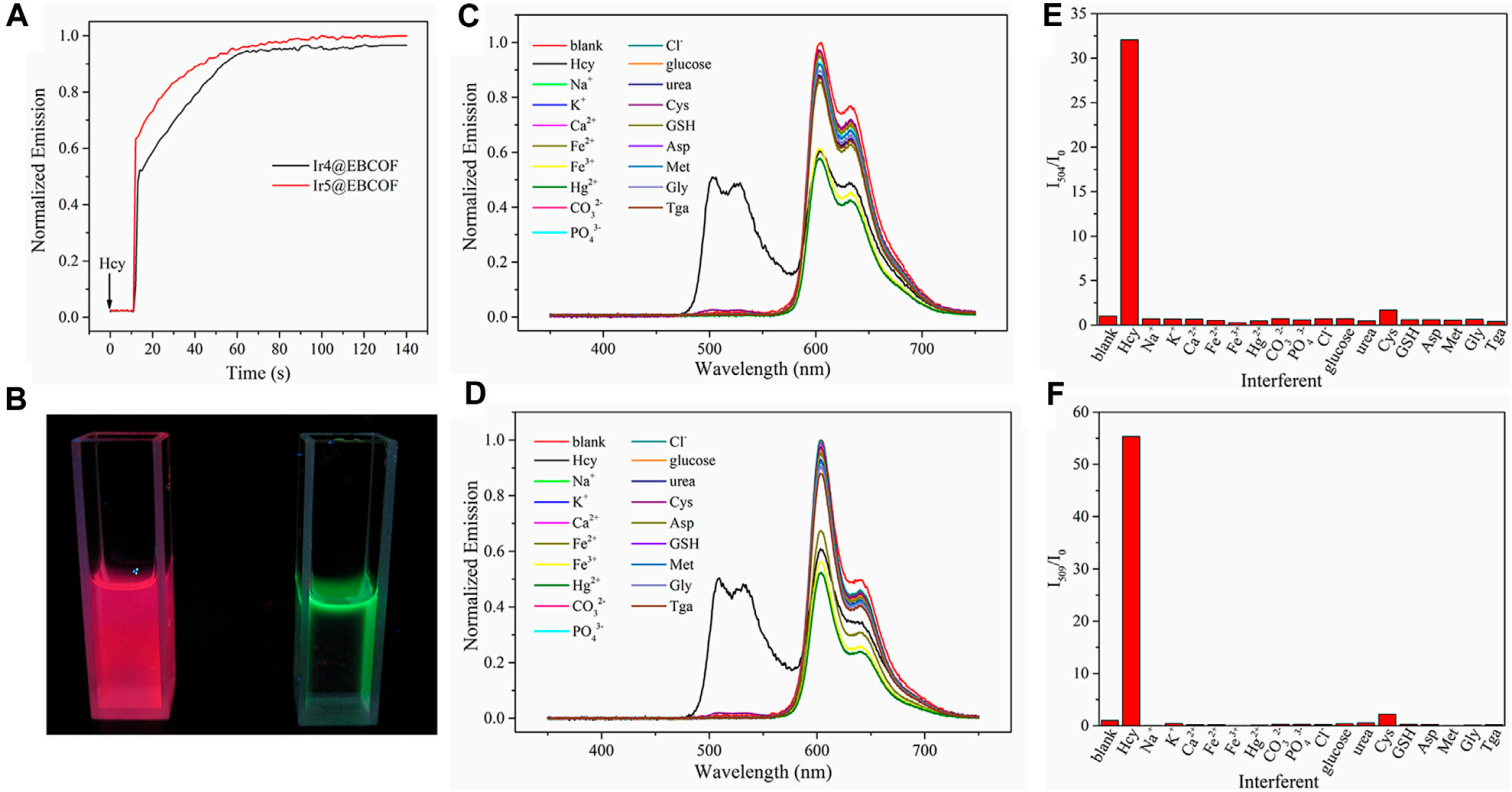
**(A)** Emission intensity monitoring of Ir4@EBCOF and Ir5@EBCOF after addition of Hcy (50 μM). **(B)** Ir5@EBCOF in PBS (2.5 mg/mL) before (left) and after (right) addition of Hcy (100 μM). Emission spectra of **(C)** Ir4@EBCOF and **(D)** Ir5@EBCOF in PBS (2.5 mg/mL) in the presence of Hcy and interferents (50 μM). **(E)** I_504_/I_0_ of Ir4@EBCOF and **(F)** I_509_/I_0_ of Ir5@EBCOF in PBS (2.5 mg/mL) in the presence of Hcy and interferents (50 μM).

We next investigated the dynamic sensing performances of the probes by monitoring their emission intensity for the optimal Ir4/5@EBCOF upon gradual addition of Hcy (Figure 5A). The emission intensity was enhanced rapidly in the first 50 s and then remained smooth until saturation. The response time, defined as the time to reach 95% of the maximum emission intensity, is about 56 s for Ir4@EBCOF and 88 s for Ir5@EBCOF. During this interval, the emission color changes from red to green (Figure 5B).

A practical sensing platform always entails an inevitable situation where the analyte is dispersed in a complex environment full of competing species and interferents. According to literature (Xiong et al., 2010; Liu et al., 2012; Wang et al., 2021), most chemical sensors struggle to distinguish between Hcy and Cys molecules owing to their similar molecular structures, which means that Cys is an important interferent in Hcy sensing. However, when reacting with Cys, Ir4/5@EBCOF shows different dynamic sensing performance than Hcy. After adding Cys, the emission intensity of Ir4/5@EBCOF remained nearly constant over the initial 600 s, and the reaction time is over 25 min, which is much longer than that of Hcy (Supplementary Figure S4). When the sample was treated in an ultrasonic bath for 10 min, Ir4/5@EBCOF completed their sensing process for Hcy and reached the maximum emission intensity, and the presence of excess Cys did not affect the detection of Hcy (Supplementary Figure S5), indicating good resistance to interference at the appropriate reaction time. With a sonication time of 10 min, the sensing selectivity of Ir4/5@EBCOF was evaluated in the presence of Na^+^, K^+^, Ca^2+^, Fe^2+^, Fe^3+^, Hg^2+^, CO_3_^2-^, PO_4_^3-^, Cl^−^, glucose, urea, Cys, GSH, aspartic acid (Asp), methionine (Met), glycine (Gly), and thioglycolic acid (Tga) (Figures 5C–F). As seen in the figure, although the emissions of Ir4/5@EBCOF at 604 nm exist in the presence of all the interferents, the emissions at 504 nm/509 nm appear only in the presence of Hcy. These results clearly indicate the good selectivity of the proposed probe to Hcy.

Based on the encouraging sensing performance, we carried out practical experiments on human serum samples to detect Hcy. For the control experiments, PBS solutions with different Hcy concentrations were adopted, and the recovery values were calculated by recording the emission spectra of Ir4/5@EBCOF in different solutions. As shown in Table 3, satisfactory recovery (96.06%–105.83%) values indicate the good sensing performances of the probes. When Hcy concentrations are lower than 40 μM, positive sensing errors are observed. As the Hcy concentration increases further, negative errors are observed. It is assumed that for higher Hcy concentrations, some of the Hcy molecules are adsorbed and trapped by the EBCOF matrix, leading to the negative sensing errors.

## 4 Conclusion

In summary, this study reports five Ir(C^∧^N)_2_(N^∧^N)^+^ compounds with various substituents, including -CHO and electron-donating -NH_2_ groups, for Hcy sensing based on investigations of the geometric structures and photophysical parameters. In addition, the mechanism of Hcy detection based on the cyclization reaction between the -CHO moiety and Hcy is proposed and verified; the degree of blue shift is also noted to be influenced by the C^∧^N ligand substituent. The optimal Ir(C^∧^N)_2_(N^∧^N)^+^ compounds, namely, Ir4 and Ir5, show obvious blue shifted emissions (by ∼100 nm) and slightly enhanced emission yields (∼10%). When Ir4 and Ir5 were doped into EBCOF via covalent bonding, the probes Ir4/5@EBCOF show good sensing performances and selectivity to Hcy. A comparison between Ir5@EBCOF and other probes on Hcy is listed in Table 4, where the comprehensive advantage of Ir5@EBCOF is observable through its wide linear dynamic range of 0.77–100 μM, low LOD of 0.23 μM, and quick response of 88 s at 25°C. Moreover, the developed probe was employed to determine Hcy in human serum samples with excellent recoveries (96.06%–105.83%). Finally, the opposing trend of dual emission intensity changes shows the feasibility of visual detection of Hcy. Thus, we expect that this work will be useful to the design of novel probes based on Ir(III) complexes.

**TABLE 4:** Key performance parameters of Ir5@EBCOF and other probes from literature.

Detection method	Materials	Linear range (μM)	LOD (μM)	Response time (min, 25°C)	Ref.
Colorimetry	Fe^3+^-TMB	2–24	2.09	10	Lin et al. (2019)
Electrophoresis separation	CE-UV	2–20	0.7	11.9	Kubalczyk et al. (2014)
Electrochemical probe	DNBSAP	6–120	4.67	10	Mostafa et al. (2022)
Spectroscopic sensing	Tyr-CDs	5–50	3.5	15	Zhu et al. (2018)
Spectroscopic sensing	SQM-NBD	0–70	0.072	20	Wang et al. (2022)
Spectroscopic sensing	FA-NCD	0–50	2.276	/	Anju et al. (2023)
Spectroscopic sensing	TAT-probe	0–100	6.51	20	Su et al. (2020)
Spectroscopic sensing	Ir5@EBCOF	0.77–100	0.23	∼1.47	This work

## Data Availability

The original contributions presented in the study are included in the article/Supplementary Material, and any further inquiries may be directed to the corresponding author.
