# Supplementary material for: Phosphorescent iridium (III) complex with covalent organic frameworks as scaffolds for highly selective and sensitive detection of homocysteine
Source: Front Chem. 2024 Jun 5;12:1399519. doi: 10.3389/fchem.2024.1399519 (PMC11186017; doi:10.3389/fchem.2024.1399519)

Supplementary Material

**Synthetic details of Irn compounds (n=1~5)**

Ir1, yellow solid, yield: 41%. ^1^H NMR (DMSO-*d*_6_, 300 MHz) δ 8.71 (dd, *J* = 7.7, 1.3 Hz, 2H), 8.29 (dd, *J* = 7.9, 1.4 Hz, 2H), 7.94 (dt, *J* = 7.7, 1.5 Hz, 4H), 7.87 (td, *J* = 7.7, 1.2 Hz, 2H), 7.69 (dd, *J* = 7.6, 1.2 Hz, 2H), 7.60 (ddd, *J* = 8.5, 7.6, 1.2 Hz, 2H), 7.27 (td, *J* = 7.5, 1.3 Hz, 2H), 7.16 (td, J = 7.6, 1.3 Hz, 2H), 7.05 (td, J = 7.4, 1.5 Hz, 2H), 6.96 (ddd, J = 9.7, 7.2, 1.3 Hz, 2H), 6.40 (dd, J = 9.7, 1.5 Hz, 2H). 13C NMR (DMSO-d6, 75 MHz) δ 154.71, 152.65, 152.62, 150.83, 150.80, 149.13, 148.67, 145.18, 145.13, 137.16, 137.14, 132.81, 132.77, 132.26, 132.23, 129.73, 129.71, 129.08, 129.06, 127.41, 127.39, 125.10, 125.08, 125.05, 124.57, 124.53, 121.79, 121.77.

Ir2, yellow solid, yield: 34%. 1H NMR (DMSO-d6, 300 MHz) δ 9.94 (d, J = 0.9 Hz, 2H), 8.71 (dd, J = 7.7, 1.3 Hz, 2H), 8.35 (d, J = 2.1 Hz, 2H), 8.29 (dd, J = 8.0, 1.5 Hz, 2H), 7.99 – 7.81 (m, 4H), 7.73 (ddd, J = 19.3, 7.9, 1.6 Hz, 4H), 7.60 (ddd, J = 8.5, 7.6, 1.3 Hz, 2H), 7.27 (td, J = 7.6, 1.4 Hz, 2H), 7.16 (td, J = 7.6, 1.4 Hz, 2H), 6.91 (d, J = 8.1 Hz, 2H). ^13^C NMR (DMSO-*d*_6_, 75 MHz) δ 189.27, 189.25, 155.85, 155.29, 154.06, 154.03, 150.80, 150.77, 149.18, 148.69, 137.82, 137.79, 137.40, 137.37, 132.81, 132.77, 132.26, 132.23, 129.08, 129.06, 127.72, 127.70, 127.41, 127.39, 126.24, 126.18, 125.10, 125.08, 121.78, 121.76.

Ir3, yellow solid, yield: 35%. ^1^H NMR (DMSO-*d*_6_, 300 MHz) δ 9.94 (d, J = 0.7 Hz, 2H), 8.53 (dd, J = 7.0, 2.0 Hz, 2H), 8.39 – 8.25 (m, 4H), 7.98 – 7.81 (m, 6H), 7.73 (ddd, J = 19.3, 7.9, 1.6 Hz, 4H), 7.16 (td, J = 7.6, 1.4 Hz, 2H), 6.91 (d, J = 8.1 Hz, 2H), 6.70 (s, 2H). ^13^C NMR (DMSO-*d*_6_, 75 MHz) δ 189.27, 189.25, 155.34, 155.29, 154.06, 154.03, 148.66, 148.65, 145.47, 143.89, 137.82, 137.79, 137.39, 137.36, 135.00, 132.26, 132.23, 131.35, 127.72, 127.70, 127.41, 127.39, 126.24, 126.18, 125.82, 125.36, 121.78, 121.75.

Ir4, yellow-red solid, yield: 31%. ^1^H NMR (DMSO-*d*_6_, 300 MHz) δ 9.94 (d, J = 0.8 Hz, 2H), 8.39 – 8.25 (m, 6H), 7.87 (td, J = 7.6, 1.2 Hz, 2H), 7.81 – 7.65 (m, 4H), 7.16 (td, J = 7.6, 1.4 Hz, 2H), 6.99 (d, J = 2.2 Hz, 2H), 6.91 (d, J = 8.1 Hz, 2H), 6.59 (dd, J = 10.9, 2.2 Hz, 2H), 5.87 (s, NH). ^13^C NMR (DMSO-*d*_6_, 75 MHz) δ 189.27, 189.25, 155.85, 155.29, 154.06, 154.03, 152.13, 152.10, 150.28, 150.25, 149.71, 148.66, 137.82, 137.79, 137.39, 137.36, 132.26, 132.23, 127.72, 127.70, 127.41, 127.39, 126.24, 126.18, 121.78, 121.75, 116.28, 109.78, 109.75.

Ir5, yellow-red solid, yield: 30%. ^1^H NMR (DMSO-*d*_6_, 300 MHz) δ 9.94 (d, J = 0.7 Hz, 2H), 8.39 – 8.25 (m, 6H), 7.96 – 7.81 (m, 4H), 7.81 – 7.65 (m, 4H), 7.49 (t, J = 7.7 Hz, 2H), 7.28 (s, 4H), 7.16 (td, J = 7.6, 1.4 Hz, 2H), 6.91 (d, J = 8.1 Hz, 2H). ^13^C NMR (DMSO-*d*_6_, 75 MHz) δ 189.27, 189.25, 155.34, 155.29, 154.06, 154.03, 148.65, 146.58, 137.90, 137.82, 137.79, 137.40, 137.36, 136.41, 132.26, 132.23, 127.72, 127.70, 127.41, 127.39, 126.24, 126.18, 126.14, 124.37, 123.78, 121.78, 121.75.

**Supplementary Figure 1.** ^1^H NMR titration of Ir4 with Hcy (1:0, 1:1 and 1:2).


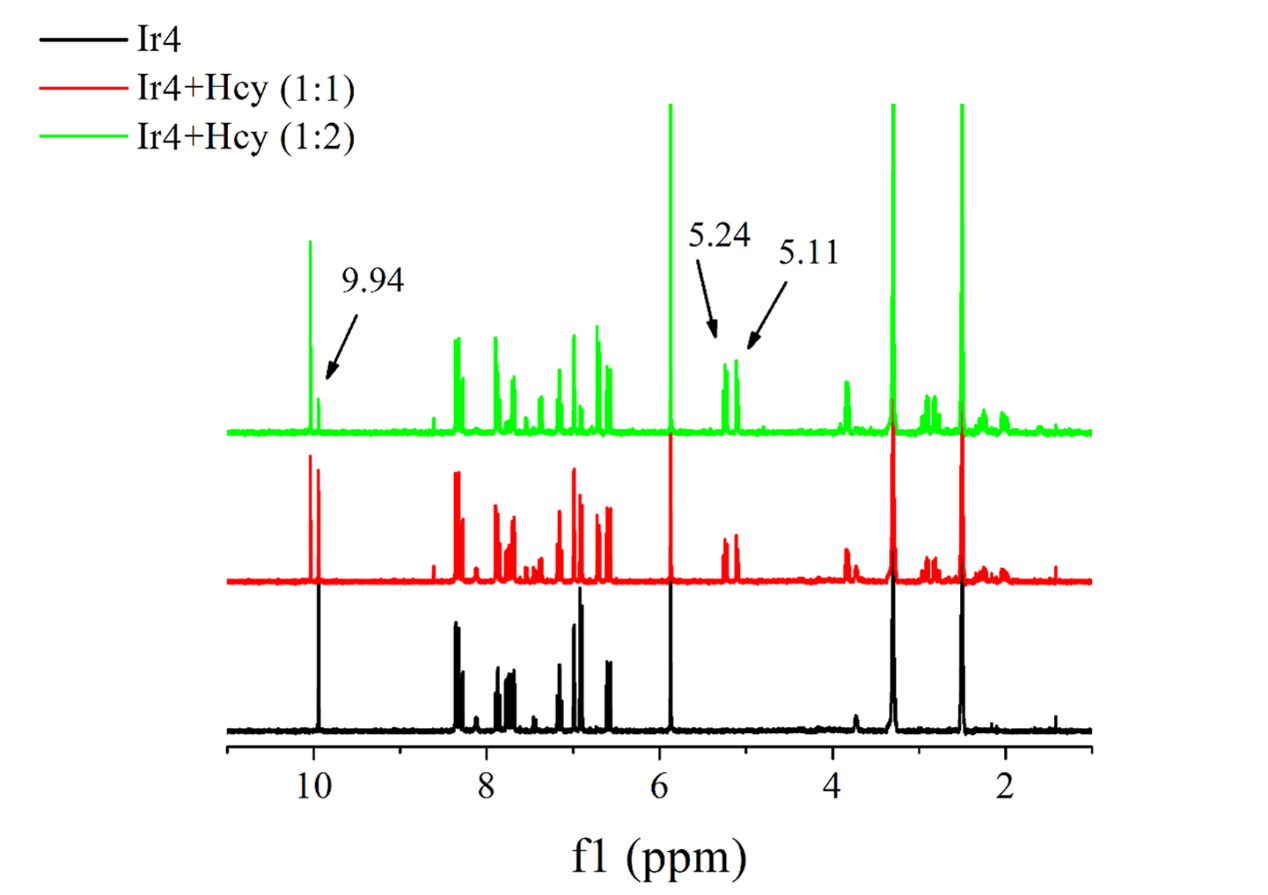


**Supplementary Figure 2.** High-resolution ESI-mass spectrum of Ir4 (**A**) and Ir5 (**B**) after the addition of Hcy.


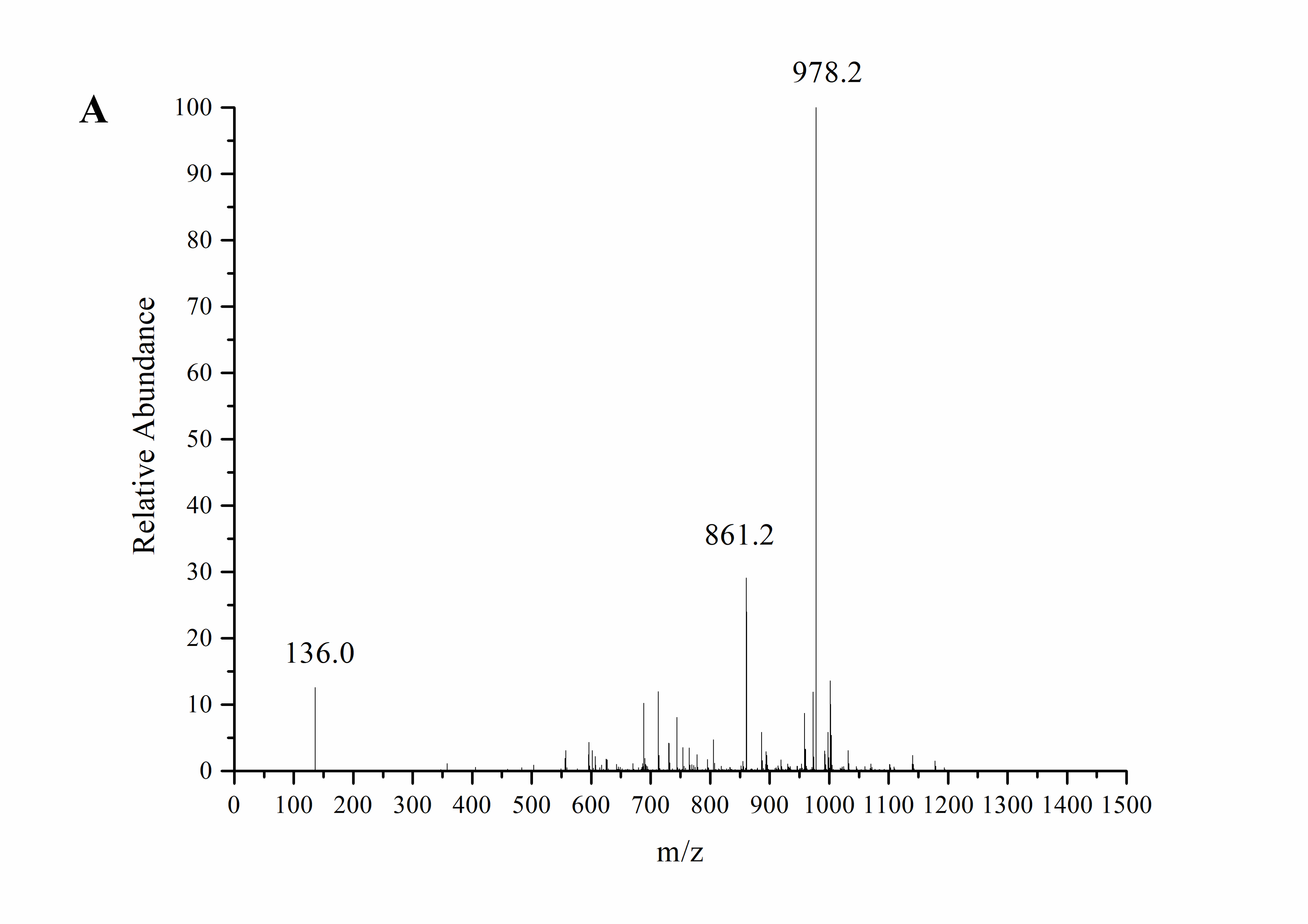

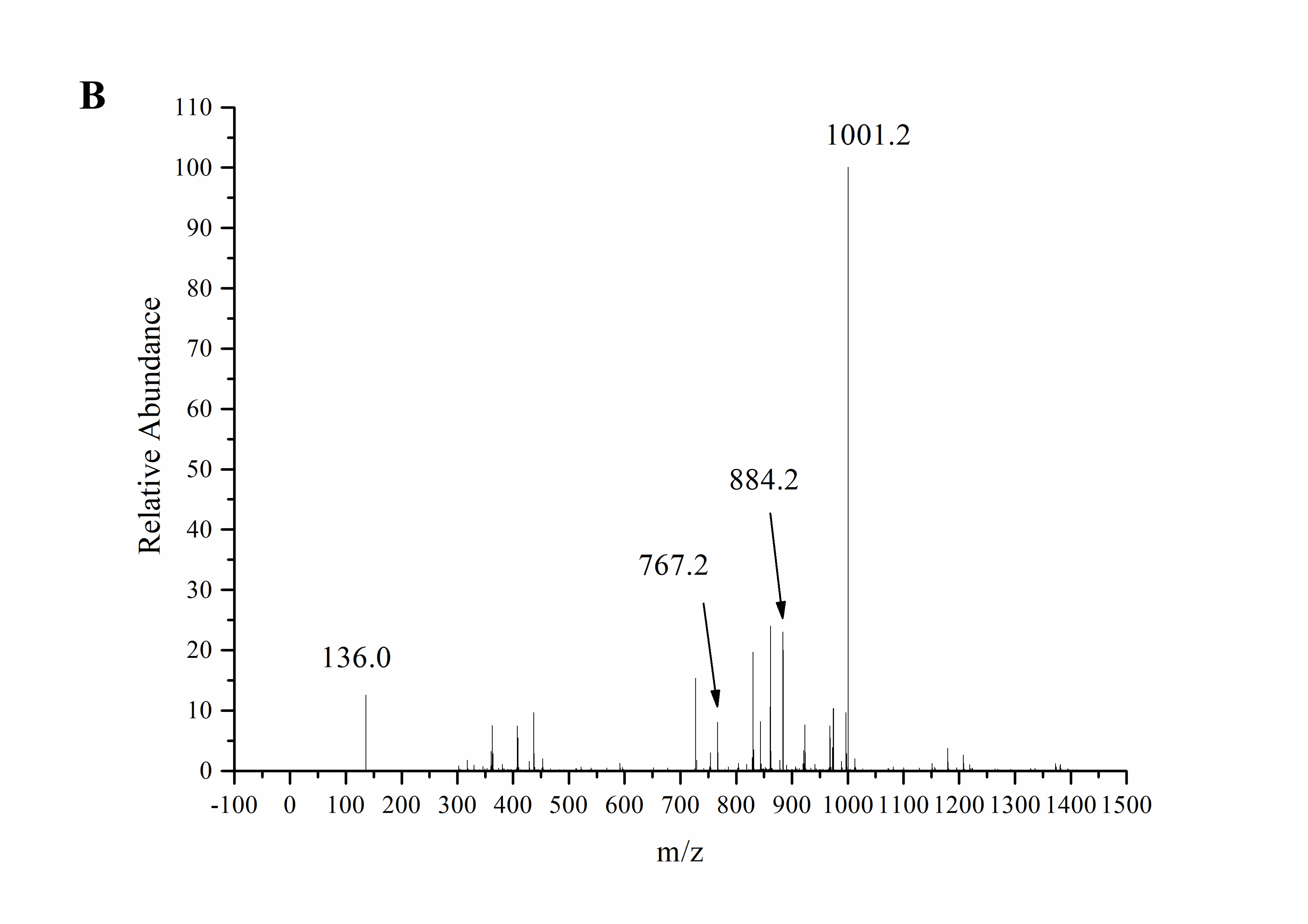


**Supplementary Figure 3.** The molecular size of Ir4 (diameter~1.2 nm).


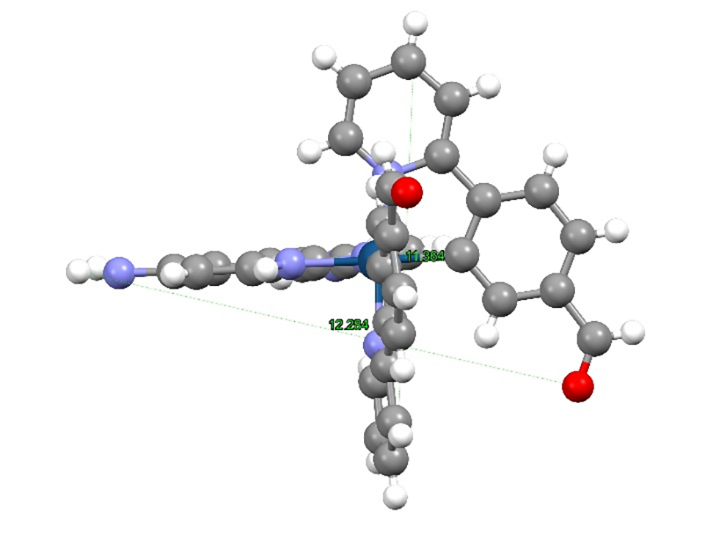


**Supplementary Figure 4.** Emission intensity monitoring of Ir4@EBCOF and Ir5@EBCOF after adding Cys (50 μM).


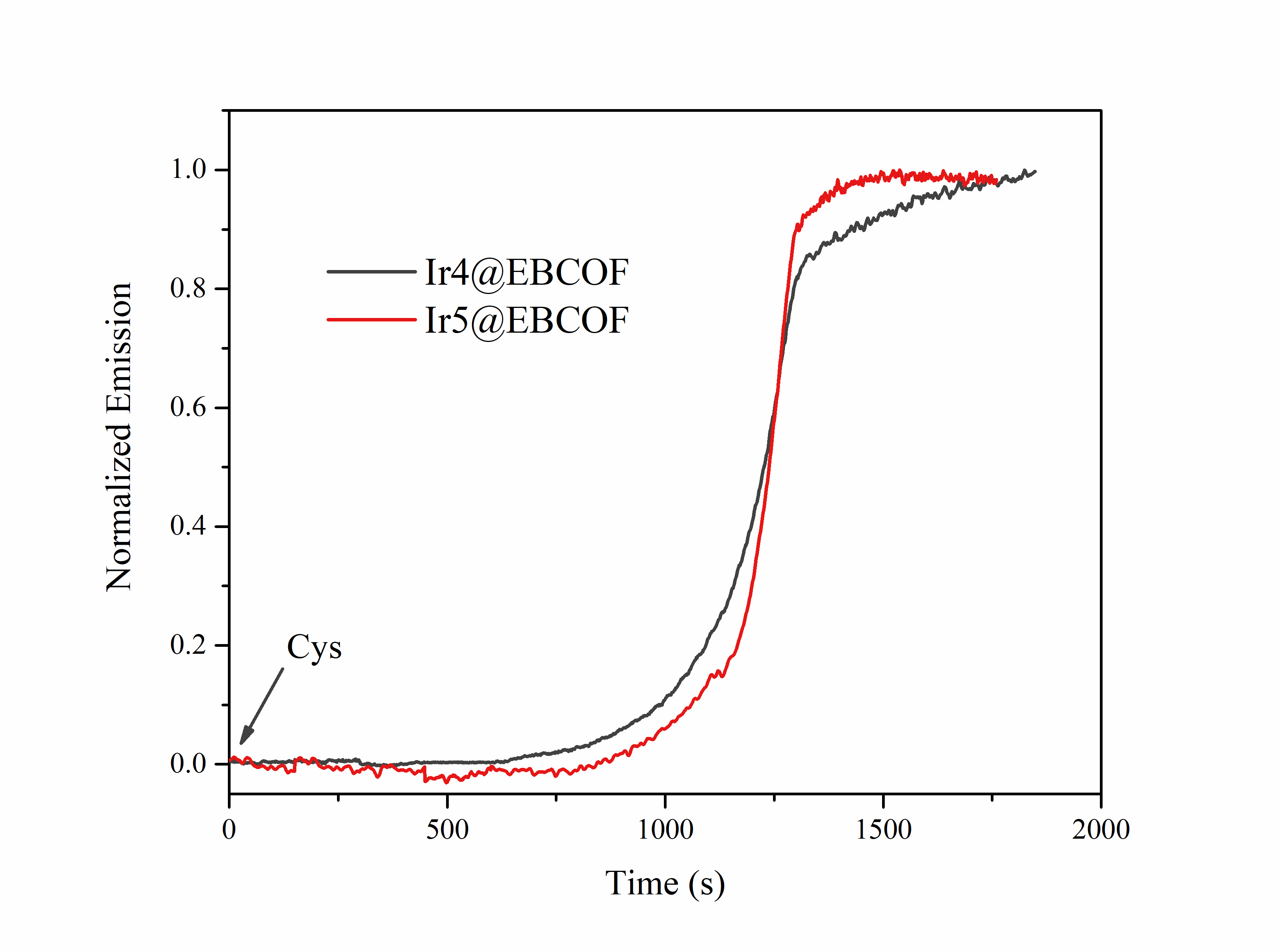


**Supplementary Figure 5.** Emission spectra of Ir4@EBCOF (**A, B**) and Ir5@EBCOF (**C, D**) in PBS (2.5 mg/mL) for Hcy (50 μM) analysis treated with different times of ultrasonic bath, in the presence of Cys (100 μM) or not.


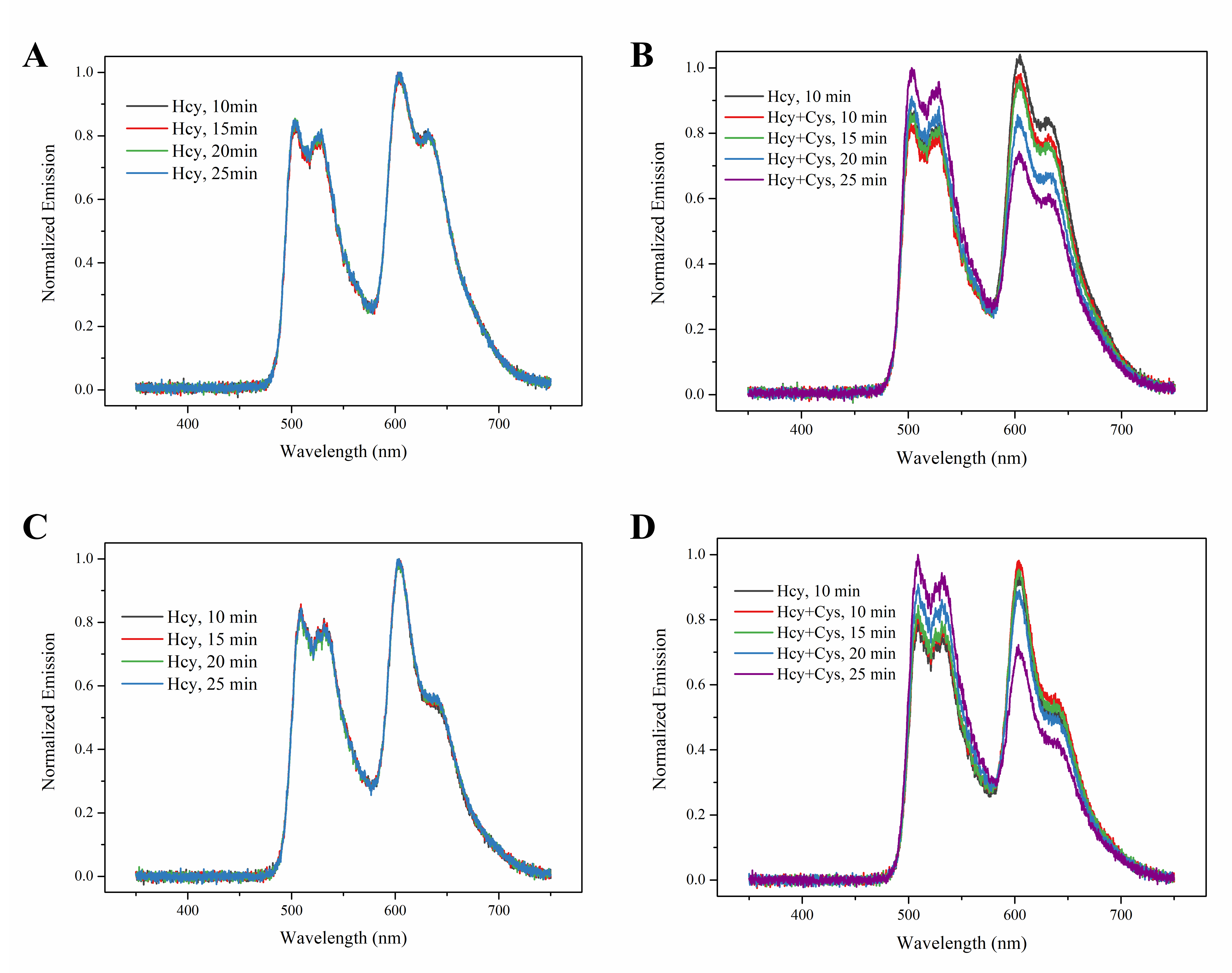


**Supplementary Figure 6.** ^1^H NMR of Ir1.


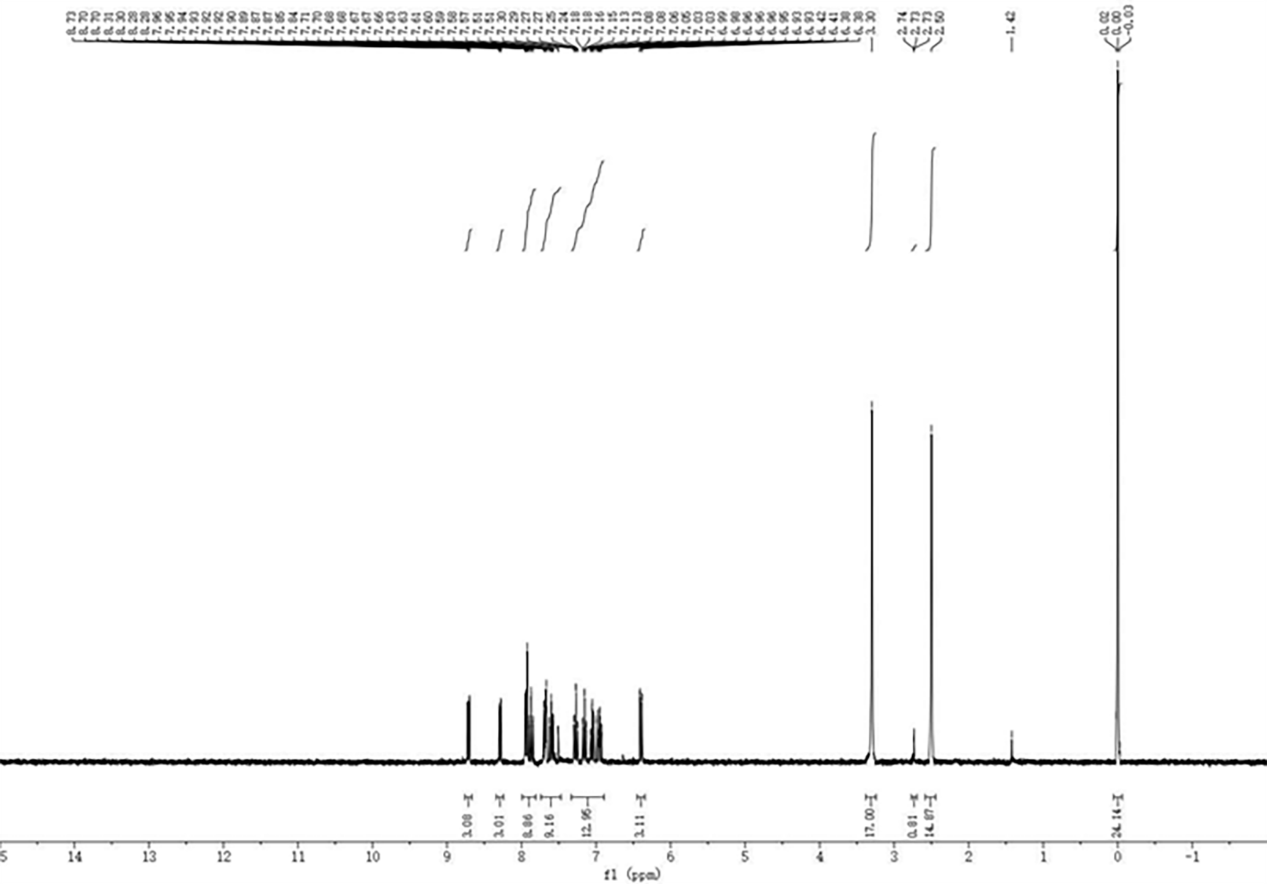


**Supplementary Figure 7.** ^1^H NMR of Ir2.


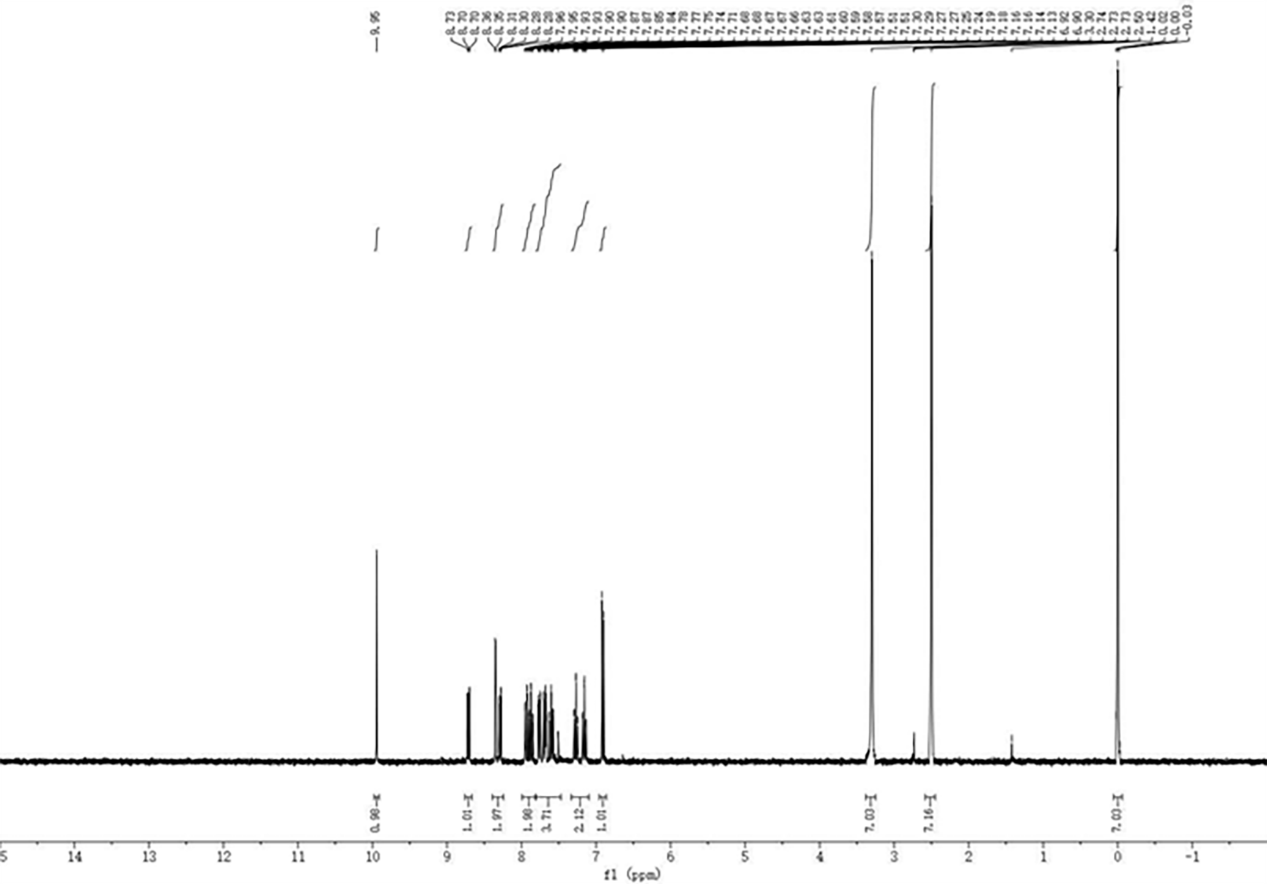


**Supplementary Figure 8.** ^1^H NMR of Ir3.


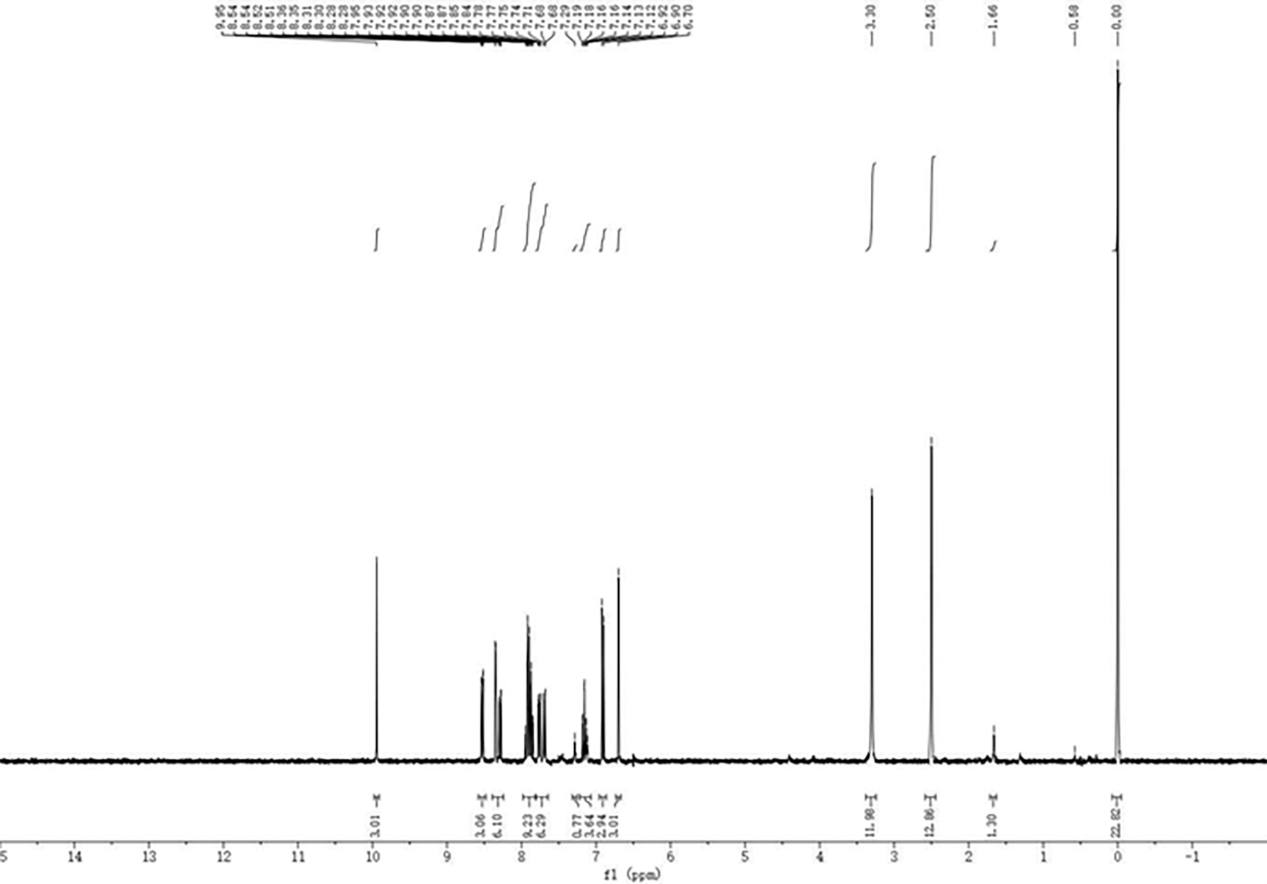


**Supplementary Figure 9.** ^1^H NMR of Ir4.


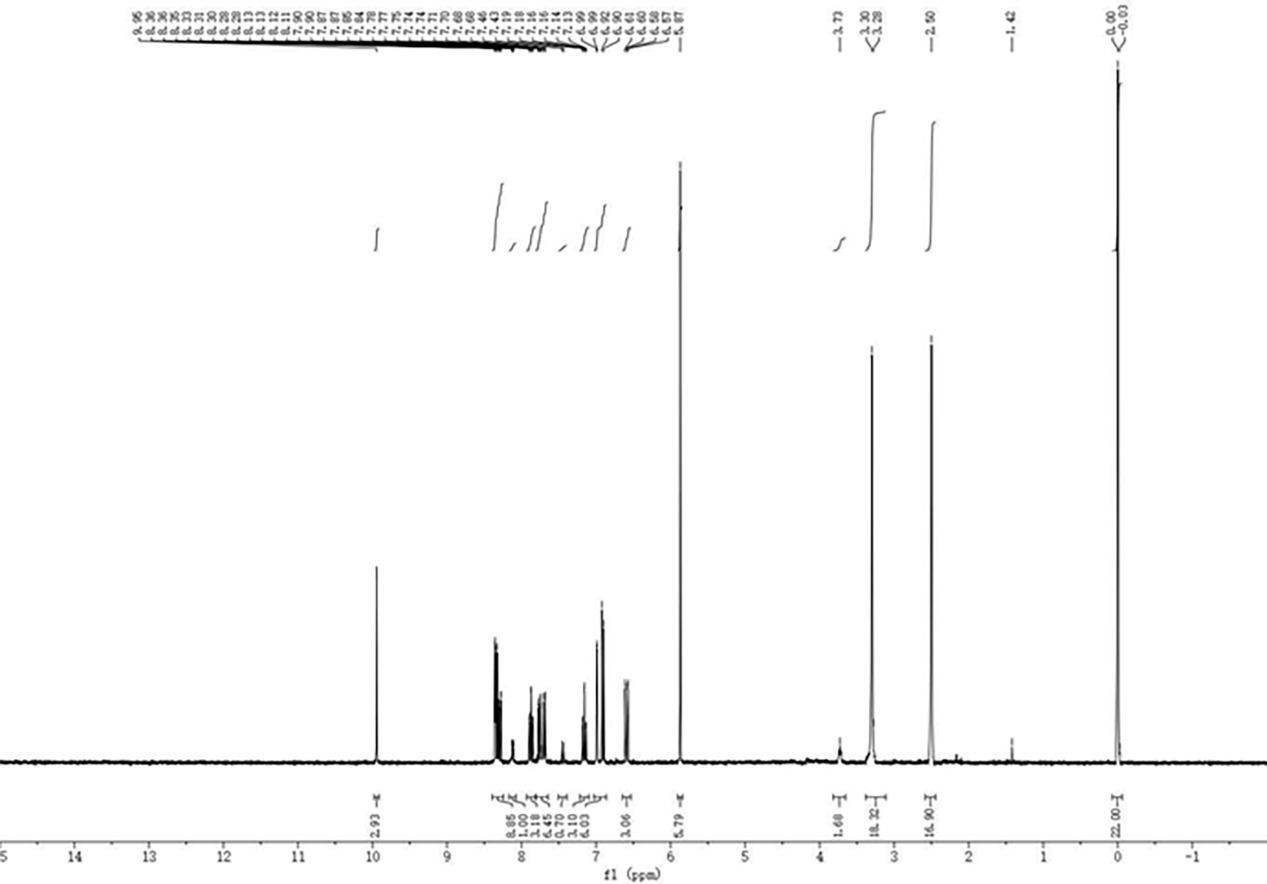


**Supplementary Figure 10.** ^1^H NMR of Ir5.


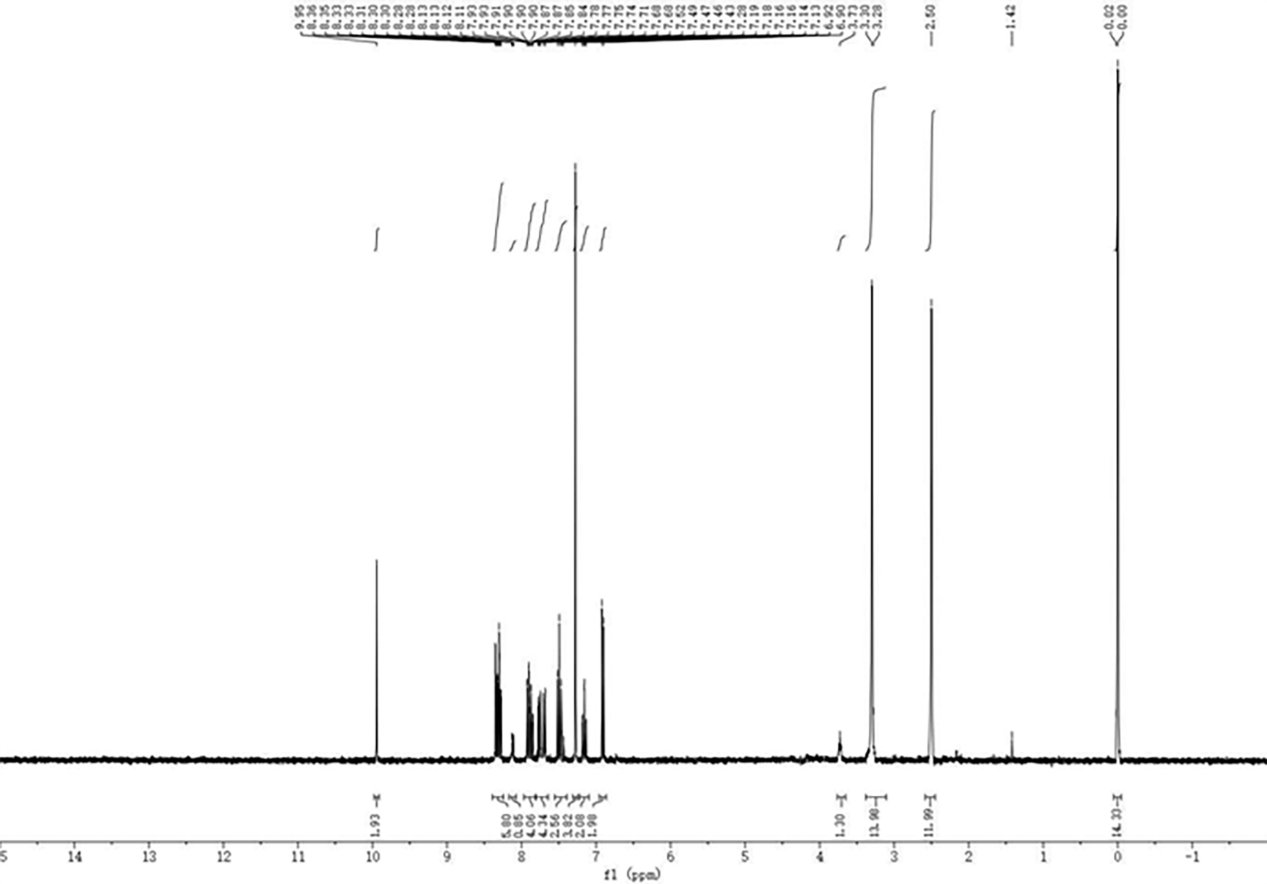

Supplement: Supplementary file 1 [file DataSheet1.docx]
